# A Cell State Monitoring System with Integrated In Situ Imaging and pH Detection

**DOI:** 10.3390/s23239340

**Published:** 2023-11-22

**Authors:** Zening Li, Rongtao Zhang, Fangliang Xu, Jian Yang, Lin Zhou, Hongju Mao

**Affiliations:** 1 State Key Laboratory of Transducer Technology, Shanghai Institute of Microsystem and Information Technology, Chinese Academy of Sciences, Shanghai 200050, China; lizening211@mails.ucas.ac.cn (Z.L.); xufangliang23@mails.ucas.ac.cn (F.X.); zhoulinzlw@mail.sim.ac.cn (L.Z.); 2 Center of Materials Science and Optoelectronics Engineering, University of Chinese Academy of Sciences, Beijing 100049, China; 3 State Key Laboratory of Component-Based Chinese Medicine, Tianjin University of Traditional Chinese Medicine, Tianjin 301617, China; 18864651799@163.com (R.Z.); yang.j2017@tjutcm.edu.cn (J.Y.); 4 Haihe Laboratory of Modern Chinese Medicine, Tianjin 301617, China

**Keywords:** cell culture, biochip, mini-microscope, pH sensor, in situ monitoring

## Abstract

Cell models are one of the most widely used basic models in biological research, and a variety of in vitro cell culture techniques and models have been developed recently to simulate the physiological microenvironment in vivo. However, regardless of the technique or model, cell culture is the most fundamental but crucial component. As a result, we have developed a cell culture monitoring system to assess the functional status of cells within a biochip. This article focuses on a mini-microscope made from a readily available camera for in situ continuous observation of cell growth within a biochip and a pH sensor based on optoelectronic sensing for measuring pH. With the aid of this monitoring system, scientists can keep an eye on cell growth in real time and learn how the pH of the culture medium affects it. This study offers a new approach for tracking cells on biochips and serves as a valuable resource for enhancing cell culture conditions.

## 1. Introduction

Biological and chemical experiments can be controlled with the microfluidics technique, which only needs a few tens or hundreds of nanoliters of samples or reagents to mix, separate, and carry out other tasks [1]. With the continuous development of micro- and nanofabrication technology, the concept of a microfluidic chip has been proposed, which makes it possible to manipulate fluids in tens of micrometer channels [2]. As a result, a biochip is created via integrating a microfluidic chip with biotechnology. Its purpose is to manipulate and analyze a small amount of a sample to obtain a large amount of biological information. PDMS is a good material for biochip fabrication and is widely used in biomedical MEMS because of its good biocompatibility and permeability [3].

Traditional in vitro cell culture has been widely used for metabolism and toxicity studies of drugs, exogenous substances, and chemicals. In order to simulate the physiological micro-environment in vivo, numerous methods and models, such as two-dimensional culture [4,5], perfusion culture [6], multicellular co-culture [7,8], and organ-on-a-chip [9,10], have been developed recently. The fact that cells can proliferate normally is a prerequisite for these models to be used further. Therefore, a cell culture assay system must be created in order to evaluate new models.

During the construction of biochips, the cells inside the chip are usually observed to characterize their physiological functional state, and the common method is to observe the growth of the cells under the microscope [11,12]. However, there are two problems with observation in the current biochip culture systems. First, the cells in the chip need to be cultured in a cell incubator under constant temperature and constant CO_2_ concentration, and the traditional observation method is to remove the chip from the incubator and place it under a large microscope for observation. This method will change the external environment in which the cells are located and may introduce contaminants such as bacteria, which may affect the functional state of the cells. Second, the microscope is large and difficult to integrate into the cell culture incubator, and the traditional observation method requires the introduction of too many manual operations if one wants to observe the cells and the color of the culture medium for several times, which is unable to accomplish the continuous real-time monitoring of the cells. The strong demand has led to the development of products from Leica [13], Zeiss, Olympus [14], Nikon [15], and others [16], and there are already solutions for placing the imaging platform in the culture unit. The Provi CM20 (Olympus), one of the more representative products, places the monitoring head and the cell culture in an incubator, where the cells are counted and the level of fusion is determined at regular intervals, enabling remote monitoring of cell culture [14] It enables remote cell culture monitoring. However, most of these products are only suitable for regular culture devices such as flasks or Petri dishes and have limited ability to detect customized biochips and require specific hardware and software systems. In addition, optical coherence tomography and quantitative self-interference spectroscopy were also used to monitor cell growth in biochips [17,18]. Greenbaum et al. performed real-time monitoring of neonatal cardiomyocyte beats following treatment with drugs such as Adriamycin and isoprenaline with a lens-free imaging system [19]. Wei et al. combined an external lens with a mobile phone into a compact imaging system for imaging and length quantification of single molecule DNA strands [20]. In order to visualize the removal of endogenous epithelium and local delivery of exogenous cells in situ, Kim et al. developed a customized micro optical imaging device integrated with a bioreactor [21]. This device has shown great potential in space cell imaging, but the system complexity is high and is not suitable for simple cell culture monitoring. In order to solve the above problems, a mini-microscope created by Zhang et al. enabled the observation of fibroblasts and 16 μm polystyrene microspheres [22]. However, the device still needs to have its flexibility and focusing capabilities increased.

The pH value of the culture medium is one of the most crucial parameters because a cell culture is usually extremely strict in terms of nutritional requirements and environmental factors. It reflects the growth rate of the cells, the CO_2_ concentration in the environment, and the presence or absence of bacterial contamination, as well as affecting the transmembrane transport of substances, the synthesis of the extracellular matrix, and other functions [23,24,25]. Although the pH range of 7.2–7.4 is generally considered to be a physiologically comfortable range for most human cells, the pH might differ for other cells or in certain unique circumstances [26,27,28]. To balance the impact of cellular metabolites on pH, conventional cell culture environments must maintain 5% CO_2_, generating bicarbonate in the medium. But, as the products accumulate up, this controlling ability diminishes, resulting in an unbalanced pH of the culture medium, which can adversely affect cell growth. Therefore, it is necessary to find an effective means of monitoring the pH inside the biochip.

The most typical way to measure pH in cell culture is through visual inspection, and, due to phenol red being added to the medium, the color of the medium changes as the pH changes [29]. The human eye may not be able to recognize the difference between this color change in biochip systems due to the relatively small volume of solution inside the chip. Another popular monitoring technique relies on potentiometric pH sensors based on metal oxides. These sensors are easy to fabricate and integrate, but their accuracy depends on the stability of the reference electrode [30]. In addition, long-term interaction with a liquid environment may change the culture medium or even the cells. Li et al. proposed a pH monitoring method based on machine vision technology as artificial intelligence advanced [31]. In order to achieve non-invasive pH monitoring, they established a mathematical relationship between the pH and the HSV model by extracting the cytosol’s color properties from the culture plate. However, the method is only effective when the sample color differs noticeably from the reference color, and, in biochip systems, there are frequently only a few microliters of culture medium present in the chamber, which fails to satisfy the requirements. To address these problems, a variety of microfluidic-based optical detection technologies have been developed due to their high capability in analyzing low volumes of liquids [32,33,34]. Most importantly, they do not necessitate the connection of electrodes inside the reactor, greatly reducing the possibility of biological contamination.

In this study, we developed a biochip culture monitoring system with integrated in situ imaging and pH measurement modules for monitoring the functional status of cells in the chip in real time. There are three primary components to the system: (1) a biochip for cell culture, (2) a mini-microscope for in situ imaging of cells, and (3) a sensor to detect the pH of the culture medium. In order to assess the chip’s potential for in situ monitoring, we first used the mini-microscope to observe and record the growth of cells within 24 h of inoculation. Next, we extended the incubation time of the cells in the microarray to 96 h and measured the pH value of the cell culture solution at different time points. Finally, we compared and analyzed the obtained pH results with the cell images at the corresponding times.

## 2. Materials and Methods

### 2.1. Biochip Design and Manufacturing

The chip was fabricated using a standard soft lithography process. The pattern on the chip was designed using CAD 2020, and then the pattern was transferred to the wafer using 250 μm thick SU-8 photoresist. A four-inch wafer was first baked at 170 °C for 15 min to keep the surface dry. The wafer was then spin-coated with a 2100 negative photoresist at 500 rpm for 10 s with 100 rpm/s acceleration and 1100 rpm for 50 s with 300 rpm/s acceleration, resulting in a 250 μm photoresist film. Subsequent wafer handling is described in detail in previous work [35]. The mixed PDMS was degassed in a vacuum chamber for 1 h to remove air bubbles. The treated PDMS was poured onto the prepared wafers and cured on a hot plate at 80 degrees for 90 min. The inlets and outlets were then punched at the designated locations using a 1.4 mm diameter hole punch. The above PDMS layer was bonded to a thin glass cover chip (0.13–0.16 mm thick, Citotest Scientifice, Nanjing, China) via oxygen plasma treatment. The chip is shown in Figure S1, with a cavity volume of approximately 20 μL.

### 2.2. Mini-Microscope Design and Construction

**Figure 1:**
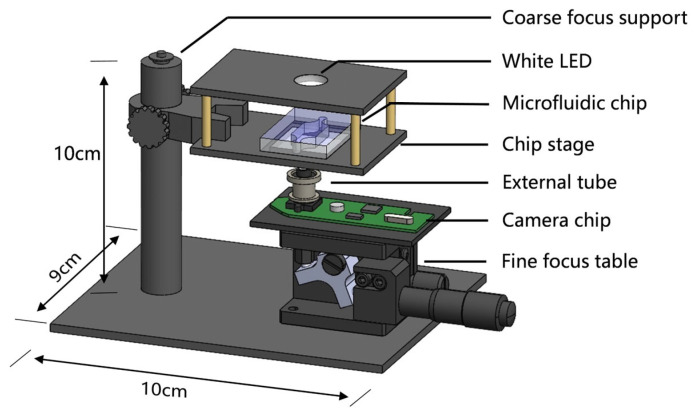
Schematic structure of the mini-microscope platform.

We modified the commercially available Logitech C270i camera (Resolution 720 p, Pixels 900,000) based on this principle by first flipping the camera’s lens so that the lens’s role becomes to magnify the object image. In optics, we know that the size of the image is related to the distance between the lens and the CMOS sensor; the greater the distance, the larger the image. Therefore, the external tube was created using 3D printing to achieve precise control of the distance. Next, a linear fit between the target magnification and the length of the external receiver was carried out. The length of the external tube was calculated according to the target magnification to achieve the personalized design of the magnification. To facilitate the focusing observation of the cells inside the chip, the circuit board of C270i was fixed on an optical stage (LZ40, Huike, Cangzhou, China) to perform focusing and imaging at different magnifications. A 1 W white LED light bead was integrated above the chip carrier stage for illumination during observation (Figure 1 and Figure S2a). Finally, in order to improve the ability to monitor the functional state of the cells in the chip, the program developed in the computer was configured with the function of calling the camera connected to the computer, and the functions of the image display, photo taking, as well as video recording were written through the specially designed user interface, which, in combination with the pH detection module, constitutes an integrated control system for monitoring the cells in the chip.

### 2.3. Fabrication of pH Sensor

#### 2.3.1. System Principle and Design

**Figure 2:**
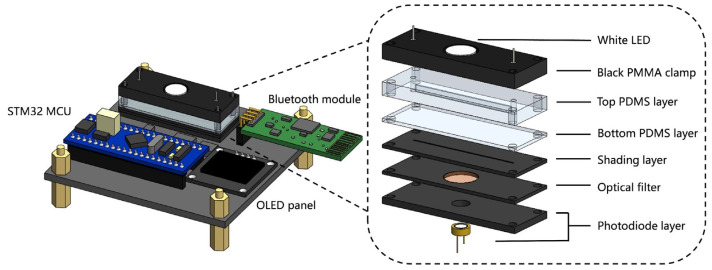
Schematic structure of the pH sensor and exploded view of the core detection section.

The pH sensor is made up of a photodiode, an LED, a polymethylmethacrylate (PMMA) holder, a pH detection chip, a filter, and a detection circuit (Figure 2, Figure S2b and Figure S3). To send a light signal to the sensor, a white LED worked as the light source. The medium containing phenol red (peak absorption wavelength: 560 nm) was passed from the inlet to the pH detection chip to absorb the light. A long pass filter (LPF510, cut-on wavelength: 510 nm, Shanghai Mega-9 Optoe-lectronic Co., Ltd., Shanghai, China) was added above the photodiode because the absorbance of phenol red varies with pH and is more pronounced at wavelengths longer than 510 nm [34]. The detection circuit is designed based on the STM32 minimum system board, which was shown in Supplementary Information. To actually make the MCU and computer separate, eliminating the intricate wires to the PC, a Bluetooth module (HC-06) is additionally added to the detection circuit for wireless data transmission, and a miniaturized Li-ion battery module is used to power the minimal system board. These modifications help to miniaturize and simplify the detection system.

#### 2.3.2. Detection Chip Design and Fabrication

The pH detection chip is made from polydimethylsiloxane (PDMS). The inverted mold was first modeled in 3D in Solidworks 2021. The model was then imported into a 3D printer (Formlabs Form 3+), and the print material (resin, Clear V4) was added. To remove the resin from the surface, the printed molds were soaked in isopropyl alcohol (IPA) for an entire night. The model was cleaned sequentially with ethanol absolute and DI water and then placed in the UV curing chamber at 40 °C for 8 h. PDMS was prepared as previously described, poured into the treated molds, and cured in an oven at 65 °C. After 4 h, the chips were removed and carefully peeled from the molds with tweezers. This step prepared the top layer of the chip, which contained microfluidic channels with a width of 500 μm and a height of 2 mm. The same procedure is used to prepare the chip’s bottom layer, which is without any structure. The two PDMS layers were bonded together via oxygen plasma treatment. The chip was then placed in an oven at 65 °C for 30 min to enhance the bonding effect.

### 2.4. Computer Program Design for Control of the Monitoring System

To facilitate the control of the mini-microscope and pH sensor, we developed a QT-based application on the computer to control the mini-microscope and the interaction with STM32. In order to implement the camera call, OpenCV must first be configured in the QT development environment. The personalized UI is used to program the functions of image displaying, photo and video recording, etc. The data transmission between the pH sensor and the computer is performed through serial communication, and the sensor is controlled by the computer by sending the specified character string. In addition, to visualize the change in pH over a period of time, the time and pH information transmitted to the computer is processed by a third-party library function (Qcustomplot) for real-time plotting and displayed in the UI.

### 2.5. In-Chip Cell Culture

**Figure 3:**
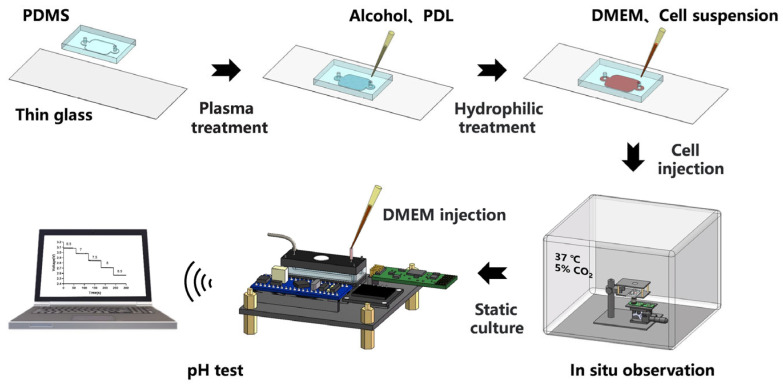
Schematic diagram of the biochip preparation process and monitoring system for cell culture.

BEAS-2B cells (provided by Procell Life Science& Technology Co., Ltd., Wuhan, China) were used for the experiments, and the complete medium used consisted of Dulbecco’s modified Eagle’s medium (DMEM), basal medium, 10% fetal bovine serum, and antibiotics (1% penicillin and 1% streptomycin). Culture flasks for inoculated cells and microchamber culture chips were kept in a 37 °C incubator containing 5% CO_2_ concentration. Cells were first proliferated and grown in cell culture flasks at a density of 70–80%; cells were dissociated using sterile phosphate-buffered solution (PBS) and 0.1% trypsin; after centrifugation at 1000 rpm for 4 min, the cell precipitate was resuspended in a cell suspension using 1 mL of complete DMEM medium; and the cell density of the suspension was calculated as 2.3 × 10^6^ cells ml^−1^. Before inoculating cells, the biochip must be treated. The prepared chip must first be sterilized by external UV sterilization and internal ethanol disinfection. The interior of the microchip was then coated with 0.1 mg/mL of Poly-D-lysine (PDL) and incubated at 37 °C for 30 min [36]. After the incubation was completed, the PDL inside the chip was replaced with sterile PBS and washed. Finally, an amount of cell suspension mixed with complete medium was aspirated according to the bottom surface area of the prepared biochip, and the cells were injected into the biochip to with cell density of 6.0 × 10^5^ cells cm^−2^. Once the cell inoculation was complete, the inlet and outlet of the bioreactor were plugged with PDMS sticks, and the culture system was placed in an incubator (Figure 3).

## 3. Results

### 3.1. Characterization of the Mini-Microscope

We calibrated the miniature microscope’s magnification in order to confirm the precise relationship between it and external tube length. The specific method is to first measure the width of the field of view of the microscope (Olympus, Tokyo, Japan) at 4×, 10×, and 20× with an accuracy of 0.1 mm and approximately 2.50 mm, 1.00 mm, and 0.50 mm, respectively, which was verified to be found to satisfy the equation:

(1) $$\frac{Magnification\;1}{Magnification\;2}=\frac{Magnification\;2\;field\;of\;view\;width}{Magnification\;1\;field\;of\;view\;width}$$

**Figure 4:**
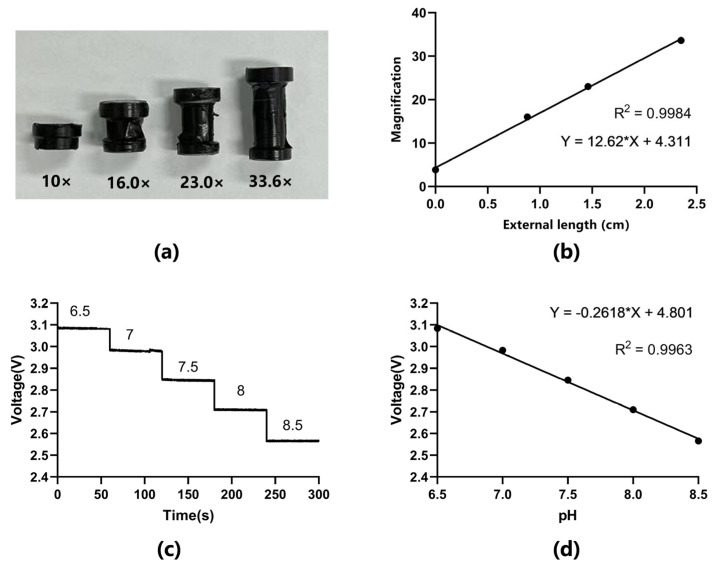
(**a**) Preparation of the external tube corresponding to different magnifications by 3D printing. (**b**) Fitting curve for the relationship between magnification and external tube length (R^2^ = 0.9984). (**c**) Voltage values corresponding to pH 6.58.5. (**d**) Fitting curve of pH 6.5–8.5 with its corresponding voltage (sensitivity: 261.8 mV/pH, R^2^ = 0.9963).

Afterwards, the webcam was measured using the same method, the initial test field of view width was measured to be 2.6 mm without the external tube connected, and the initial magnification of the lens was calculated to be about 3.85× by Equation (1). It can be inferred that the magnification has the same linear relationship with the length of the external tube since the magnification is proportional to the distance from the lens to the CMOS sensor. For this reason, 3D printing technology was also used to create different lengths of external tubes, which can be tightly connected to the CMOS sensor housing and lens at both ends. A total of three sets of external tubes were fabricated, with lengths of 8.8 mm, 14.6 mm, and 23.5 mm, and the magnification was calculated in the same way, and the results were 16.0×, 23.0×, and 33.6×, respectively (Figure 4a). Linear fitting of the four sets of data obtained showed a high linear relationship (Figure 4b, R^2^ = 0.9984). Therefore, by accurately controlling the length of the external receiver, we can customize the magnification of the mini-microscope to meet different observation needs. There was no operation to change the magnification during the in situ monitoring of cells. In this study, we selected 10× as the magnification and prepared the corresponding external tube in order to better observe the entire process of cell differentiation from inoculation to value-added differentiation.

### 3.2. Calibration of pH Sensor

In order to characterize the pH sensor, we first configured the medium with pH values of 6.5, 7, 7.5, 8, and 8.5 and measured the pH using a commercially available pH meter (PHS-25). The culture media with different pHs were then passed into the pH detection chip separately using a syringe pump. The white LED transmitted different light intensities through the various pH media as a result of the phenol red’s light-absorbing properties. To ensure the validity of the measurement, we provide a stable voltage source and take the same lead turn-on time during the experiment. Due to the black PMMA fixture in the device limiting the entry of ambient light, pH measurement does not need to be carried out under dark conditions. The converted voltage values were read in real time through an STM32 and sent to a specially developed computer program for simultaneous curve plotting (Figure 4c). Voltage and pH have a very strong linear relationship, according to linear fitting of the five sets of data (Figure 4d, R^2^ = 0.9963). So, we conclude that the validity pH range of the sensor is 6.5–8.5. Finally, the fitted relation was imported into the computer program to display the measured pH values synchronously.

### 3.3. 24 h Continuous In Situ Observation of Cells in Biochip

**Figure 5:**
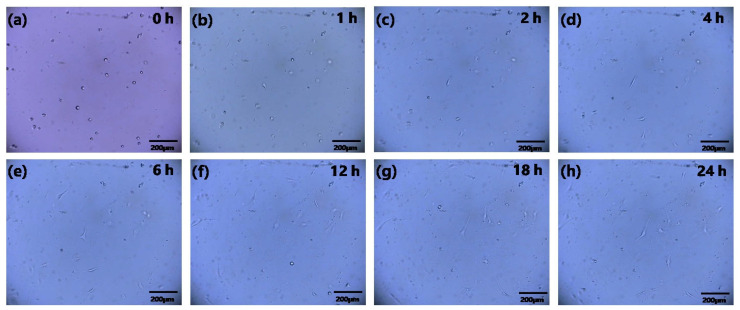
A total of 24 h of mini-microscope observation of BEAS-2b cell growth. (**a**–**h**) various time points for cell growth. Scale bar, 200 μm.

In this section, we performed one 24 h cell culture experiment. The time period of 24 h was chosen here to observe the process of the cells from inoculation to complete apposition, with the main purpose of verifying the feasibility of the mini-microscope for continuous monitoring. We inoculated the cell suspension into the PDL treated chip, combining the mini-microscope with cell culture, and successfully constructed an in situ real-time monitoring system for cells. Figure 5 shows that when the cells were first inoculated, they were suspended in a spherical shape. After 2 h of static culture, the cells’ adhesion proteins increased, and it was possible to see that the cells started to gradually attach to the wall, changing from spherical to flat type. After 4–6 h of the attaching period, the majority of the cells had successfully attached to the wall. The images taken at various times between 6 and 24 h revealed that the cells had started to proliferate and differentiate. The change in cell position in the images may be caused by the change in cell state, which resulted in the cells deviating from the originally focused imaging plane. Images of the BEAS-2b cell growth process demonstrated that the mini-microscope enables real-time monitoring of the entire cell growth process.

### 3.4. pH Test of Cell Culture Medium in Chip

**Figure 6:**
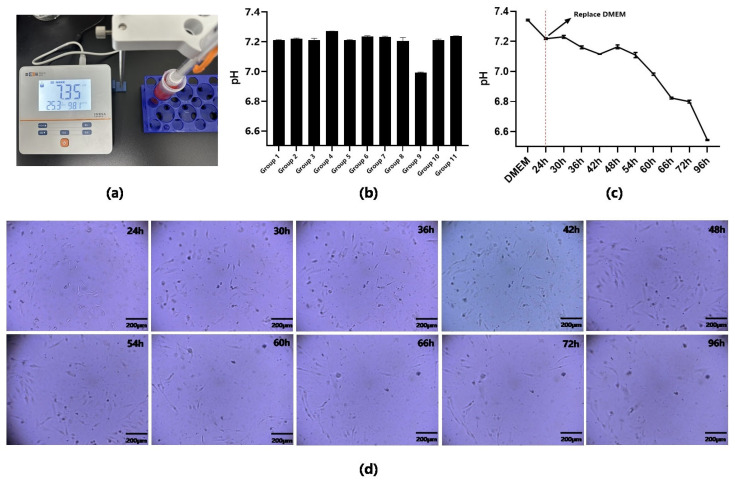
Calibration of the pH sensor and the detection of the pH value of the culture medium in biochip. (**a**) Commercialized pH detector for calibration of pH sensors and verification of DMEM pH value. (**b**) Detection results of pH sensor on parallel groups pH after 24 h of cell culture. (**c**) Change curves of the pH value of the cell culture medium in biochip at different time. (**d**) Real-time observation of cell growth status corresponding to pH. Scale bar: 200 μm.

In this section, we used a cost-effective pH detection model to track changes in pH during cell culture. The accuracy of the pH sensors was compared prior to the formal experiment, and the first set of data in Figure 6c shows the pH value of the culture medium (pH ≈ 7.34) measured with the pH sensor, which is essentially consistent with the measurement results of the commercially available pH meter (pH ≈ 7.35) in Figure 6a. The pH sensor used in this study still maintains excellent accuracy under conditions of low cost, easy operation, and low detection consumption.

We prepared 11 groups of biochips as described in Section 2.5 and inoculated the cells at a density of 6.0 × 10^5^ cell cm^−2^. When the cells were cultured to the 24th hour, the culture media were aspirated, and pH was measured separately. The PDMS sticks at the inlet and outlet of the chip were first removed, and the culture medium inside the chip was aspirated by a 20 µL pipette gun. Afterwards, the volume of the pipette gun was adjusted to remove the air from the front of the tip. Finally, the culture medium is injected into the pH test chip through the external Teflon hose to complete the pH measurement. The pH values of the 11 groups of chips at the 24th hour are displayed in Figure 6b. With the exception of the fourth and ninth groups, the pH values of other groups are relatively close. After observing the two groups of cells under the microscope, we concluded that the rapid decrease in pH was due to the leakage of the seal at the inlet and outlet of the chip, and the slow decrease in pH was due to the poor state of the cells inside the chip and the slow adherence to the wall, resulting in the reduction in medium consumption. To ensure the scientific validity of the subsequent experiments, only the remaining model group was selected for the subsequent experiments.

Next, we replaced the culture medium for the remaining nine groups of cells after screening, continued to incubate them, and measured the pH of one of the chips sequentially every 6 h from the 24th hour until the incubation time was 72 h. The detection results of pH values in each chip are shown in Figure 6c. We can see that the pH of the culture medium had an overall decreasing trend after the medium was changed. In the 24–54 h, the pH value was always above 7.1, which is the more suitable range for cell growth, but below 7.1 after the 54th hour, which may cause cell apoptosis. Combined with Figure 6d, it can also be seen that the number and state of cells in the field of view remained good during 24–54 h, whereas the cell state changed significantly after the 54th hour, and a large number of cells began to fall off. Through the above analysis, we can conclude that with the continuous proliferation of cells in the chip, the metabolites in the culture medium continue to accumulate, leading to a continuous decline in the pH value of the culture medium. The continuous low pH culture environment is also unfavorable to cell proliferation and growth, resulting in the stagnation of the proliferation process and the gradual detachment of some cells from the adherent state. In this study, we successfully constructed a uniform experimental model group, and the pH detection system used in the experiment was able to accurately respond to micro changes in pH in the culture medium.

## 4. Discussion

In traditional cell culture models, cells are usually grown and differentiated in culture flasks or Petri dishes. The advantage of this approach is that the culture system is large and capable of capacity for more cell growth and high-volume cell proliferation. However, a large capacity often means more resource consumption. Therefore, it is not a good choice to use the traditional models for accurate disease modelling and drug screening. Compared with the large-capacity culture system of the traditional culture model, the micro-culture of a biochip is more controllable, efficient, and sensitive. In this experiment, there was no significant difference in the growth process between cells cultured in the chip and cells cultured in the culture flasks. Although the number of cells used in the 11 groups of chips was much less than the number of cells required for culture in cell flasks, the operation of culturing cells on chips was more difficult.

Cell imaging is an important means of cell monitoring, playing a crucial role in both cell subculture and in vitro model construction. In Section 3.3, we cultured BEAS-2B within the microarray for 24 h and recorded the whole process of the cells from the suspension state to the complete adhesion. The results proved the feasibility of the homemade mini-microscope for the continuous monitoring of cell culture. As can be seen from Figure 5, in the 24 h culture monitoring experiment, the cells just went through the process from suspension to adhesion to simple proliferation, and the number of cells did not change significantly. During the continuous monitoring, we also found that, as our mini-microscope observed the cells on a fixed focal plane, the cell morphology would change over time, and some cells would “disappear” during the growth and need to be refocused in order to be observed. So, we simply displayed the process of the change in cell morphology in the form of a continuous image and did not assess the status of the cells by counting. Compared to the currently commercialized cell monitoring system, the mini-microscope is small in size, low in cost, and easy to operate. The magnification can be customized, and the package structure can be changed according to the needs of the experimenter. In addition, due to its small size and easy integration, it also has the potential to be applied to in vitro model construction. Most of the current in vitro model construction adopts dynamic culturing, which usually connects multiple sensors as well as microfluidic hoses around the chip, which requires an imaging approach that does not disrupt the overall system connectivity. Compared to optical coherence tomography [17] and quantitative self-interferometric spectroscopy [18], mini-microscopes do not require complex optical systems, making it more user-friendly for experimental operators. A variety of micro-imaging systems have been developed based on lens-free technology as well as mobile phones. Although they can monitor cell beating [19] and image DNA strands [20], these micro-imaging systems are not suitable for long-term monitoring of chip-based in situ biological systems. The device designed by Kim’s group shows great potential for spatial cell imaging [21], but the system was too complex and not suitable for 2D cell culture monitoring. Our proposed mini-microscope has advantages in terms of structure, cost, and user operation and can be well suited for in situ real-time imaging of cells within a biochip.

pH is also another indicator that reflects the state of cells. We measured the pH of the culture medium inside the chip via the pH sensor in Section 3.4. Although the pH value at the 48th hour was not lower than the previous moment as expected, this result is acceptable, considering that cell growth is not always consistent within the different chips. The overall pH value tended to decrease from 7.34 to 6.55 during the 96 h incubation process, which is consistent with our expected results. Typically, cells stop proliferating or die when the pH of the medium is less than 7.1. As can be seen from Figure 6d, a large number of cells started to shed at the 60th hour compared to the 54th hour, indicating that the cells were in an apoptotic state. It can also be verified from Figure 6c that the pH started to be less than 7.1 between the 54th and 60th hours. Compared to the electrochemical measurement of pH [30], our sensor is more bio-friendly and avoids the effect of electrode surface oxides on the culture medium. The sensor is based on photoelectric detection, which does not require particularly significant color changes to detect pH [31], so it has higher accuracy and sensitivity [32,33,34]. It can solve the problem of the inability of common commercial pH meters to measure small volumes of media with high sensitivity. In addition, as it is based on fluidic detection, it also has the potential to be integrated into in vitro modelling systems. Compared to traditional electrochemical measurements, it avoids contamination caused by electrode and solution reactions and is also more suitable for the construction of in vitro model systems.

## 5. Conclusions

In this study, a system for in-chip cell in situ real-time monitoring and culture medium pH detection was developed. This system has the advantages of low cost, simple operation, and high integration. It also overcomes some of the limitations of traditional cell observation by enabling cell state observation without changing the cell culture’s environment. Meanwhile, we also constructed a pH sensor based on photoelectric sensing, which greatly decreased the possible biological contamination during the testing process. The pH sensor also has a high sensitivity, allowing for accurate pH value detection even in a small volume. This study focuses on the system’s capacity to track the conditions of the cells during static culture. The current direction of biochip development is to accurately simulate the microenvironment of cells in vivo and in vitro, and shear force is one of the easier physical quantities to simulate, which is usually achieved by using perfusion culture. In this case, we can integrate the pH sensor and the mini-microscope together in an incubator, and drive the fluid through a micropump to achieve dynamic cell culture and real-time measurement of the pH of the medium in the system. With the development of in vitro cell culture models, the technology of culturing various cells on a chip will become an important tool for future human medical research. Via this monitoring system, scientists can track and analyze the physiological state of cells in these models in real time and respond quickly to emerging issues. In order to monitor numerous physiological indicators of cells and make it applicable to more in vitro culture models, we will integrate more sensors and control systems in our future work.

## Data Availability

Data are contained within the article.

## Supplementary Materials

The following supporting information can be downloaded at: https://www.mdpi.com/article/10.3390/s23239340/s1, Figure S1: CAD design of the biochip and its structure after bonding completion; Figure S2: Pictures of the mini-microscope and the pH sensor; Figure S3: Hardware design of the pH Sensor.
