# Supplementary material for: A Cell State Monitoring System with Integrated In Situ Imaging and pH Detection
Source: Sensors (Basel). 2023 Nov 22;23(23):9340. doi: 10.3390/s23239340 (PMC10708649; doi:10.3390/s23239340)
Supplement: Supplementary file 1 [file sensors-23-09340-s001.zip › sensors-2642967-supplementary.pdf]

Article

A Cell State Monitoring System with Integrated In Situ Imaging and pH Detection

Zening Li [1,2,†], Rongtao Zhang [3,†], Fangliang Xu [1,2], Jian Yang [3,4], Lin Zhou [1] and Hongju Mao [1,2,*]

[1] State Key Laboratory of Transducer Technology, Shanghai Institute of Microsystem and Information Technology, Chinese Academy of Sciences, Shanghai 200050, China; lizening211@mails.ucas.ac.cn (Z.L.); xufangliang23@mails.ucas.ac.cn (F.X.); zhoulinzlw@mail.sim.ac.cn (L.Z.)

[2] Center of Materials Science and Optoelectronics Engineering, University of Chinese Academy of Sciences, Beijing 100049, China

[3] State Key Laboratory of Component-Based Chinese Medicine, Tianjin University of Traditional Chinese Medicine, Tianjin 301617, China; 18864651799@163.com (R.Z.); yang.j2017@tjutcm.edu.cn (J.Y.)

[4] Haihe Laboratory of Modern Chinese Medicine, Tianjin 301617, China

* Correspondence: hjmao@mail.sim.ac.cn

† These authors contributed equally to this work.

Fig. S1 (a) shows the CAD design of the biochip; the subsequent lithography was performed according to this pattern. Fig. S1 (b) shows the bonded completed chip and the state of the cell suspension after adding cells within it.

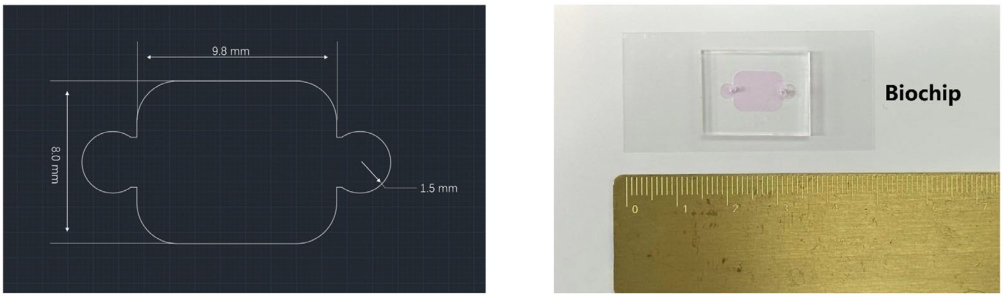

(a) (b)

Figure S1. CAD design of the biochip and its structure after bonding completion.

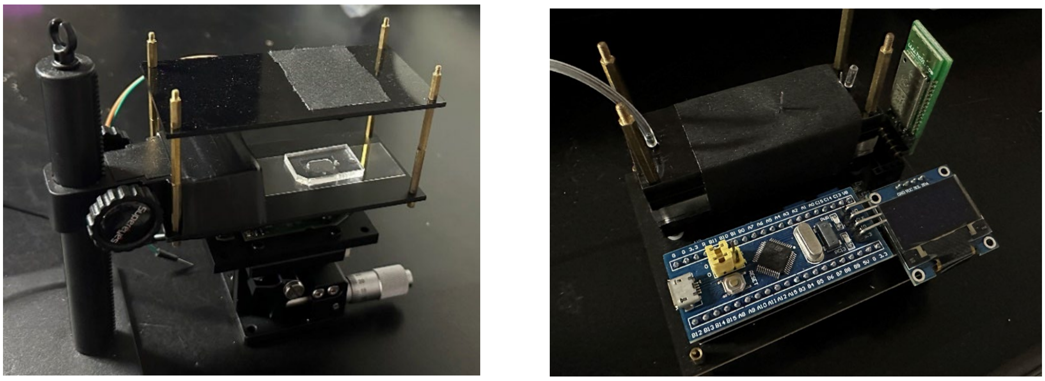

(a) (b)

Figure S2. Pictures of the mini-microscope and the pH sensor.

Fig. S3 shows the hardware circuit diagram of the pH sensor based on the STM32 minimum

system board.

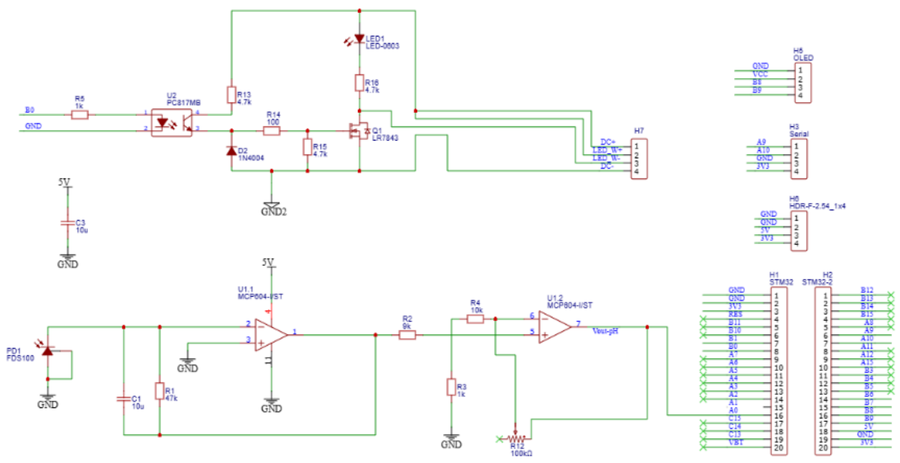

Figure S3. Hardware design of the pH sensor.

The following is the control and signal acquisition code based on the STM32 minimum system board:

```
uint16_t AD_Value_pH, Num;
int   Step = 100;
float Time;
float a[2], v[2], w[2], w1[2];
uint8_t Voltage_num_pH[12], Time_num[12], pH[12];
int LED_White_ShowFlag = 0;

int main(void)
{
    OLED_Init();
    AD_Init();
    Timer_Init();
    Serial_Init();
    LED_Init();
    OLED_ShowString(2, 5, "pH Test");
    while (1)
    {
        if(Serial_GetRxFlag() == 1)
        {
            if(strcmp((char*)Serial_RxPacket,"Start") == 0)
            {
                OLED_ShowString(2, 1, "                ");
```

```
                        while(a[0] >= 0)
                        {
                            TIM_Cmd(TIM2, ENABLE);
                          a[0] = (float)Num * Step / 1000;
                            w[0] = (float)AD_Value_pH /4095 * 3.3;
                           w1[0] = (float)(4.801 – w[0]) / 0.2618;
                          sprintf((char*)Voltage_num_pH, "%6.4f", w[0]);
                          sprintf((char*)Time_num, "%7.2f", a[0]);
                              sprintf((char*)pH, "%6.3f", w1[0]);
                          OLED_ShowString(2, 1, (char*)Voltage_num_pH);
                          OLED_ShowString(1, 1, (char*)Time_num);
                              OLED_ShowString(3, 1, "pH : ");
                            OLED_ShowString(3, 5, (char*)pH);

if(LED_White_ShowFlag == 0 && strcmp((char*)Serial_RxPacket, "LED_W_ON") == 0)
                              {
                                   LED_W_ON();
                                   OLED_ShowString(3, 1, "                    ");
                                   OLED_ShowString(3, 1, "pH-test-start");
                                    LED_White_ShowFlag = 1;
                              }
else if(LED_White_ShowFlag == 1 && strcmp((char*)Serial_RxPacket, "LED_W_OFF") == 0)
                              {
                                   LED_W_OFF();
                                   OLED_ShowString(3, 1, "                    ");
                                   OLED_ShowString(3, 1, "pH-test-end");
                                    LED_White_ShowFlag = 0;
                              }

                    }
              }

        }
   }
}

void TIM2_IRQHandler(void)
{
      if(TIM_GetITStatus(TIM2, TIM_IT_Update) == SET)
      {
            Num ++;
            Time = Num * Step / 1000;
            AD_Value_pH = AD_GetValue(ADC_Channel_0);
          AD_Value_O2 = AD2_GetValue_Average();
```

```
        TIM_ClearITPendingBit(TIM2, TIM_IT_Update);
        Serial_SendString("!");
        Serial_SendString((char*)Time_num);
        Serial_SendString(" ");
        Serial_SendString((char*)Voltage_num_pH);
        Serial_SendString(" ");
        Serial_SendString((char*)pH);
        Serial_SendString("#");

    }
}
```

The following shows the PC program developed based on Qt, which is used for controlling hardware and data analysis:

```
MainWindow::MainWindow(QWidget *parent)
    : QMainWindow(parent)
    , ui(new Ui::MainWindow)
{
    ui->setupUi(this);
    mIsOpen = false;
    mSend_pH = false;
    QList<QSerialPortInfo> SerialPortInfo = QSerialPortInfo::availablePorts();
    int count = SerialPortInfo.count();
    for(int i=0; i<count; i++)
    {
        ui->CboxSerialPort->addItem(SerialPortInfo.at(i).portName());
    }
    connect(&mSerialPort, SIGNAL(readyRead()), this, SLOT(on_SerialPort_readyRead()));
    timer = new QTimer(this);
     CameraFlag = 0;
    connect(timer,SIGNAL(timeout()),this,SLOT(readFrame()));
    connect(ui->Open,SIGNAL(clicked()),this,SLOT(on_Open_clicked()));
    connect(ui->Close,SIGNAL(clicked()),this,SLOT(on_Close_clicked()));
    ui->myCustomPlot2->addGraph();
    ui->myCustomPlot2->graph(0)->setPen(QPen(Qt::blue));
    ui->myCustomPlot2->graph(0)->setName("pH measure");
```

```
    ui->myCustomPlot2->legend->setVisible(true);
    ui->myCustomPlot2->xAxis->setLabel("Time");
    ui->myCustomPlot2->yAxis->setLabel("pH");
    ui->myCustomPlot2->xAxis->setRangeLower(0);
    ui->myCustomPlot2->xAxis->setRangeUpper(X2_max);
    ui->myCustomPlot2->yAxis->setRange(6.5, 8.5);
    ui->myCustomPlot2->setInteractions(QCP::iRangeDrag | QCP::iRangeZoom |
QCP::iSelectPlottables);
}
MainWindow::~MainWindow()
{
    delete ui;
}
void MainWindow::on_Open_clicked()
{
    CameraFlag = 0;
    cap.open(1);
    timer->start(10);
}
void MainWindow::on_Close_clicked()
{
    timer->stop();
    cap.release();
    ui->Camera->clear();
}
void MainWindow::readFrame()
{

    cap>>src_image;
    if(CameraFlag == 0)
    {
        QImage img = MatImageToQt(src_image);
        ui->Camera->setPixmap(QPixmap::fromImage(img));
    }
    else
    {
            QImage img = MatImageToQt(src_image);
            ui->Camera->setPixmap(QPixmap::fromImage(img));

            write.write(src_image);
    }
}
QImage MainWindow::MatImageToQt(const Mat &src)
{
```

```
    if(src.type() == CV_8UC3)
    {
        const uchar *pSrc = (const uchar*)src.data;
        Qimage qImage(pSrc,src.cols,src.rows,src.step,QImage::Format_RGB888);
        return qImage.rgbSwapped();
    }
    else
    {
        QImage qimg((const unsigned char*)(src.data), src.cols, src.rows,
QImage::Format_Grayscale8 );
        return qimg;
    }
}
void MainWindow::on_Photo_clicked()
{
    img2 = MatImageToQt(src_image);
    if(!src_image.empty())
        {
            ui->Picture->setPixmap(QPixmap::fromImage(img2));
            ui->Picture->setScaledContents(true);
        }
}
void MainWindow::on_Save_clicked()
{
Qstring filename=QFileDialog::getSaveFileName(this,tr("Save
Image"),QDir::homePath(),tr("(*.jpg)\n(*.bmp)\n(*.png)"));
    img2.save(filename, "jpg", 100);
}
void MainWindow::on_Video_clicked()
{
    CameraFlag = 4;
        QString str = QString::number(i, 10);
        QString str2 = "E:/QT/OpenCV/Video/";
        QString str3 = str2.append(str);
        QString filename = str3.append(".avi");
        write.open(filename.toLocal8Bit().toStdString(), VideoWriter::fourcc('M', 'J', 'P', 'G'),
30.0, Size(640, 360), true);
        i++;
    }
}
void MainWindow::on_VideoClose_clicked()
{
    CameraFlag = 0;
}
```

```
void MainWindow::on_BtnStart_clicked()
{
    if(flag == 1)
    {
        ui->BtnStart->setText("Draw start");
        flag = 0;
    }
    else
    {
        ui->BtnStart->setText("Draw close");
        flag = 1;
    }
}
void MainWindow::on_BtnClear_clicked()
{
    ui->CustomPlot->graph(0)->data().data()->clear();
    arrX.clear();
    arrY.clear()
    ui->CustomPlot->replot();
    TimeCounter = 1;
}
bool MainWindow::getSerialPortConfig()
{
   Here is the regular code to get the serial port, not to be shown.
}
void MainWindow::on_SerialPort_readyRead()
{
    if(true == mIsOpen)
    {
        QByteArray recvData = mSerialPort.readAll();
        if(recvData.contains('!') | temp.contains('!'))
        {
            temp.append(recvData);
        }
        if(temp.contains('#'))
        {
            STM32data = QString(temp).mid(1,21);
            ui->TeditRecieve->append(STM32data);
            temp.clear();
        }
        XData = STM32data.mid(0,6).toDouble();
        YData = STM32data.mid(7,6).toDouble();
        YDatapH =(4.801 – Ydata) / 0.2618;
        if(flag == 1)
```

```
        {
            if(XData == 0)
            {
                arrX.clear();
                arrY.clear();
            }
            arrX.append(XData);
            arrY.append(YDatapH);
            if(XData > X_max)
            {
                 ui->myCustomPlot2->xAxis->setRangeUpper(XData);
            }
            ui->myCustomPlot2->graph(0)->setData(arrX,arrY);
            ui->myCustomPlot2->replot();
        }
    } }
void MainWindow::on_Test_Start_clicked()
{
    QString Start = "@Start\r\n";
    mSerialPort.write(Start.toStdString().c_str());
    mSend_pH = true;
}
void MainWindow::on_BtnStart_2_clicked()
{
    if(flag == 1)
    {
        ui->BtnStart_2->setText("Draw start");
        flag = 0;
    }
    else
    {
        ui->BtnStart_2->setText("Draw close");
        flag = 1;
    }
}
void MainWindow::on_BtnClear_2_clicked()
{
    ui->myCustomPlot2->graph(0)->data().data()->clear();
    arrX2.clear();
    arrY2.clear();
    ui->myCustomPlot2->replot();
}
void MainWindow::on_LED_pH_clicked()
{
```

```
    if(mSend_pH == true)
    {
        ui->LED_pH->setText("LED-pH-OFF");
        QString LED1 = "@LED_W_ON\r\n";
        mSerialPort.write(LED1.toStdString().c_str());
        mSend_pH = false;
    }
    else
    {
        ui->LED_pH->setText("LED-pH-ON");
        QString LED1 = "@LED_W_OFF\r\n";
        mSerialPort.write(LED1.toStdString().c_str());
        mSend_pH = true;
    }
}
void MainWindow::on_BtnOpen_clicked()
{
    if(true == mIsOpen)
    {
        mSerialPort.close();
        ui->BtnOpen->setText("start");
        mIsOpen = false;
        ui->CboxSerialPort->setEnabled(true);
        ui->CboxBaudrate->setEnabled(true);
        ui->CboxParity->setEnabled(true);
        ui->CboxDataBits->setEnabled(true);
        ui->CboxStopBits->setEnabled(true);
    }
    else
    {
        if(true == getSerialPortConfig())
        {
            mIsOpen = true;
            ui->BtnOpen->setText("close");
            ui->CboxSerialPort->setEnabled(false);
            ui->CboxBaudrate->setEnabled(false);
            ui->CboxParity->setEnabled(false);
            ui->CboxDataBits->setEnabled(false);
            ui->CboxStopBits->setEnabled(false);
        }
    }

}
```
